# Synthesis of a TEMPO-Substituted Polyacrylamide Bearing a Sulfonate Sodium Pendant and Its Properties in an Organic Radical Battery

**DOI:** 10.3390/polym11122076

**Published:** 2019-12-12

**Authors:** Junfeng Zhu, Ting Zhu, Huan Tuo, Wanbin Zhang

**Affiliations:** 1 Shaanxi Key Laboratory of Chemical Additives for Industry, College of Chemistry and Chemical Engineering, Shaanxi University of Science and Technology, Xi’an 710021, China; zhuting9988@163.com (T.Z.); 15029062608@163.com (H.T.); 2 Shaanxi Collaborative Innovation Center of Industrial Auxiliary Chemistry and Technology, Shaanxi University of Science & Technology, Xi’an 710021, China; zhangwanbin@sust.edu.cn

**Keywords:** nitroxyl radical polymer, poly(TEMPO-acrylamide-*co*-sodium styrene sulfonate), electrochemical properties, cathode material, organic radical battery

## Abstract

A novel nitroxyl radical polymer poly(TEMPO-acrylamide-*co*-sodium styrene sulfonate) (abbreviated as poly(TAm-*co*-SSS)) was synthesized using 4-acrylamido-2,2,6,6- tetramethylpiperidine (AATP) copolymerized with styrene sulfonate sodium (SSS). AATP was synthesized through a substitution reaction of acryloyl chloride. Meanwhile, poly(4-acrylamido-2,2,6,6-tetramethylpiperidine-1-nitroxyl radical) (PTAm) was prepared as a control sample. Then, the structures of products were characterized by nuclear magnetic resonance spectroscopy (^1^H-NMR), Fourier transform infrared spectroscopy (FT-IR), high performance liquid chromatography-mass spectrometry (HPLC-MS), differential scanning calorimetry (DSC), X-Ray photoelectron spectroscopy (XPS), and electron paramagnetic resonance (EPR), respectively. Additionally, the electrochemical impedance spectra (EIS) and the charge-discharge cycling properties were studied. The results demonstrated that the poly(TAm-*co*-SSS) with the side group of sodium sulfonate adjacent to TEMPO group exhibits a better charge-discharge cycling stability than that of the PTAm. Moreover, the charge specific capacity of the poly(TAm-*co*-SSS) is larger than that of the PTAm. Besides, the first coulombic efficiency of poly(TAm-*co*-SSS) is higher in comparison with that of PTAm. These superior electrochemical performances were ascribed to the synergistic effect of sulfonate ions group and nitroxyl radical structure, which benefits the improvement of charge carrier transportation of the nitroxyl radical polymers. Consequently, the nitroxyl radical poly(TAm-*co*-SSS) is promising for use in organic radical battery materials, based on the good electrochemical properties.

## 1. Introduction

Organic-based electrode-materials can ensure the safety and cycle performance of a lithium secondary battery, and have the characteristics of bendable deformation, high specific energy, wide operating temperature range, long storage life, small self-discharge, and no memory effect, and can be used according to requirements [1,2,3,4,5]. Charge and discharge do not reduce battery performance, among other advantages [6,7]. Various organic molecules have been examined toward obtaining organic-based electrodes, such as conducting polymers, electron-donor/acceptor molecules, metal-clusters, and organic radical molecules [8,9,10]. Radical polymers are composed of non-conjugated carbon backbones that contain pendant groups bearing reversible redox products of radical species such as nitroxide, phenoxyl, and galvinoxyl radicals. Due to the fact that radical polymers are completely amorphous and lack π-conjugation, charge transport occurs locally and through a reversible oxidation–reduction (redox) reaction mechanism, the radical polymers are expected to become a new generation of electrode materials [11,12].

As a typical radical polymer, poly(4-acrylamido-2,2,6,6-tetramethylpiperidine-1-nitroxyl radical) (PTAm) indicates that it can be used as a novel, metal-free cathode material or long-period rechargeable devices [13,14]. Moreover, the stable amphoteric nitroxide block copolymers which were synthesized by block copolymerization were promising to be used as the EPR probes for bioimaging in vivo [15]. The electrochemical properties of the PTAm have been studied to confirm its potential for application in new electrode materials [16,17]. However, although good cyclability was usually reported [18,19,20], the TEMPO-substituted polyacrylamide was generally soluble in the electrolyte, leading to loss of capacity over time [21]. Crosslinking of the polymer was then a suitable option to improve the charge-discharge cycling stability [22]. In order to improve electrical properties of organic electronic polymers as electrode materials, ion-bearing repeat units were intentionally added to the macromolecular architecture of PTMA. The ion-bearing repeat units serve as intramolecular dopants in order to enhance the transport ability of the copolymers relative to pristine PTMA [23]. Furthermore, vinylsufonic acid as a balance group was introduced to PTMA to achieve both swelling and yet insoluble properties in the aqueous electrolyte solution. The excellent charging–discharging properties of the copolymer electrode allowed application to “rocking-chair-type” electrodes [24,25]. However, to our best knowledge, there are few reports on TEMPO-substituted polyacrylamide bearing the sulfonate sodium pendant which are prepared by copolymerization and used in the field of organic radical batteries.

We extended the paradigm to the realm of radical polymers through the intentional addition of ion-bearing repeat units to the macromolecular architecture of PTAm. Then, these ion-bearing repeat units were introduced in order to enhance the charge-discharge cycling stability of the copolymers relative to pristine PTAm. This objective of this work was to modify PTAm with sodium styrene sulfonate—a reactive group—to obtain a hydrophilic polymer radical polymer poly(TAm-*co*-SSS). This would allow the polymer material to adapt to a wider range of electrolyte solutions and different application requirements in electronic devices. The structure and electrical properties of poly(TAm-*co*-SSS) were investigated. The charge-discharge properties of the prepared polymers were studied by galvanostatic charge-discharge testing. The radical polymer poly(TAm-*co*-SSS) exhibits good electrochemical lithium storage performance. We demonstrated that the novel radical polymer poly(TAm-*co*-SSS) can be used in an organic radical battery. These results highlight the ability to expand the molecular doping paradigm beyond the conjugated polymer regime and into the realm of radical polymers, such that materials can be synthesized in a ready and systematic fashion. It is vital to scale up production of the organic radical polymer materials.

## 2. Experimental

### 2.1. Materials

4-Amino-2,2,6,6-tetramethylpiperidine (98%, analytically pure), toluene (analytically pure, used after molecular sieve treatment), acryloyl chloride (98%, analytically pure), azobisisobutyronitrile (AIBN), and styrene sulfonate (analytically pure, used after recrystallization from ethanol) were bought from Aladdin Industrial Co., Shanghai, China; *m*-chloroperoxybenzoic acid (*m*CPBA, 98%, analytically pure), tetrahydrofuran(99%, analytically pure) and the remaining reagents that were of analytical grade were bought from Shanghai Macklin Biochemical Co., Ltd. (Sahnghai, China).

### 2.2. Synthesis of Poly(TAm-co-SSS) and PTAm

**Scheme 1:**
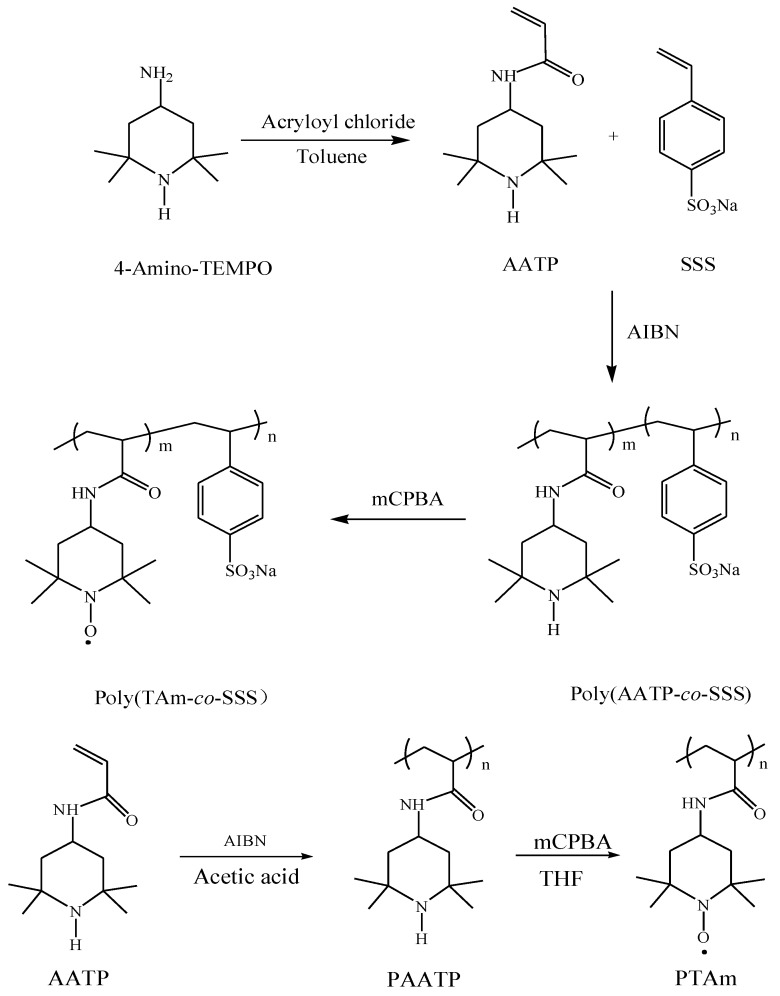
The synthesis route to poly(TAm-*co*-SSS) and PTAm.

The synthesis route of the polymer (poly(TAm-*co*-SSS)) and PTAm is shown in Scheme 1.

#### 2.2.1. Synthesis of AATP

The molecular sieve-treated anhydrous toluene (90 mL) was poured into a 100 mL round-bottomed flask, covered with a plastic stopper, and sealed with a tape. The flask was placed on a magnetic stirrer with an ice bath (T < 5 °C). The flask was pinched in the plastic plug and purged N_2_ for more than 5min. The 4-Amino-2,2,6,6-tetramethylpiperidine (4.5 mL, 0.025mol) was poured into the toluene while magnetic stirring was working. After stirring for more than 10 min, acryloyl chloride (1.9 mL, 0.022 mol) was slowly added dropwise to the flask within 30 min. Then the mixture was stirred in the ice bath for 1 h, and then at room temperature for 1 h. When the reaction was over, the resulting turbid liquid was filtered with a vacuum pump to collect a white precipitate. The precipitate was washed three times with dichloromethane, and then washed with a saturated NaHCO_3_ solution. The dichloromethane was removed by vacuum drying for 24 h. The final product was recrystallized from methanol after insoluble impurities were removed, as was water by addition of anhydrous MgSO_4_ for more than 1 h and filtration; the final product was a white crystal [26].

#### 2.2.2. Synthesis of Poly(AATP-co-SSS) and PAATP

AATP (0.2898 g, 0.001 mol) and SSS (0.2763 g, 0.001mol) (total monomer concentration of 1 mol/L, nAATP/nSSS) = 1:1) were poured into a Schlenk tube containing methanol aqueous solution (2.85 mL, methanol /water = 2:1, v/v), AIBN (0.016 g, 0.025 mmol/L) under magnetic stirring until the solid dissolved completely. The above mixed solution was frozen in liquid nitrogen. The Schlenk apparatus with a double-row tube was used to pump air through the test tube suction port. In the same time that nitrogen gas was introduced to the Schlenk tube, the frozen solution completely melted. Then, the above operation was repeated until no bubbles were generated in the solution. The test tube was purged with nitrogen, while it was placed in a 65 °C oil bath with stirring for 10 h. After that, the reaction product was poured into *n*-hexane with stirring to remove AATP, SSS, and AIBN. A white precipitate was obtained after suction filtration; then, it was dried in a vacuum oven for 24 h to remove the residual *n*-hexane. A white powder poly(AATP-*co*-SSS) was finally obtained. In the same time, radical polymer PAATP was synthesized by referring to the literature [13].

#### 2.2.3. Synthesis of Poly(TAm-co-SSS) and PTAm

The poly(AATP-*co*-SSS) (100 mg) was dispersed completely into 5 mL of tetrahydrofuran (THF) in a 100 mL three-necked flask equipped with a constant pressure separatory funnel under ultrasonic vibration. Then, *m*CPBA (200 mg) dissolved in THF (5 mL) was added into the flask through the funnel. Then, the mixed solution was stirred for 7 h in a nitrogen atmosphere. After that, the mixture was washed with 20% Na_2_CO_3_ solution. The separated organic phase solution was slowly dropped into a *n*-hexane solution to precipitate a product. Meantime, the radical polymer PTAm was synthesized according to the literature [14].

### 2.3. Characterization and Electrochemical Measurements

The monomer AATP was characterized by high performance liquid chromatography-mass spectrometry (HPLC-MS) (maXis Q-TOF, Bruker, GER). ^1^H-NMR spectra of all prepared products were recorded on a Bruker ADVANCEⅢ spectrometer (Bruker, Germany) using deuterated chloroform. FT-IR spectra were carried out on a VECTOR-22 spectrometer (Bruker, Germany) with KBr pellets at wavelength range from 500 cm^−1^ to 4000 cm^−1^. XPS measurements were taken using a AXIS X-ray photoelectron spectroscopy (AXIS Supra, Kratos, UK). Electron paramagnetic resonance (EPR) was carried out on an E500 Electron paramagnetic resonance (Bruker, Germany) at a concentration of 2 mg·mL^−1^ in tetrahydrofuran (THF) at room temperature. DSC measurements were carried out on the as prepared samples, using a Discovery DSC 250 series instrument (TA Instruments, New Castle, DE, USA). The samples were initially heated to 200 °C and cooled to −50 °C at 10 °C·min^−1^ under a nitrogen gas purge. The data shown were from the final scans from −50 °C to 200 °C at 10 °C·min^−1^.

The prepared poly(TAm-*co*-SSS) and PTAm were, respectively, mixed with acetylene black and binder (PVDF) in a mass ratio (8:1:1). Then, a small amount of *N*-methyl pyrrolidone was added dropwise into the above mixtures with stirring to form a uniform paste. The paste was placed on an aluminum foil and dried under vacuum at 70 °C for 12 h. And then, the dried paste was cut into slices as positive electrodes. CR2025 coin-type batteries were assembled using a lithium metal sheet as the counter electrode and negative electrode, l mol·L^−1^ LiPF_6_/EC+DMC (1:1) solution as an electrolyte, and celgard 2400 as the diaphragm in an argon filled glove box. The charge and discharge of the battery were tested by the BTS-XWJ-6.44S-00052 multi-channel battery program control tester (Xinwei, Shenzhen, China) at the voltage range from 2.0–4.3 V with the current density of 100 mA·g^−1^ at room temperature. The electrochemical impedance spectra of the battery were recorded on a CPARSTATMC electrochemical workstation (Princeton Applied Research, Princeton, NJ, USA) at an open circuit potential with the voltage amplitude of 10 mV and the frequency range from 100 kHz to 0.1 Hz.

## 3. Results and Discussion

### 3.1. Characterization of Monomer AATP

**Figure 1:**
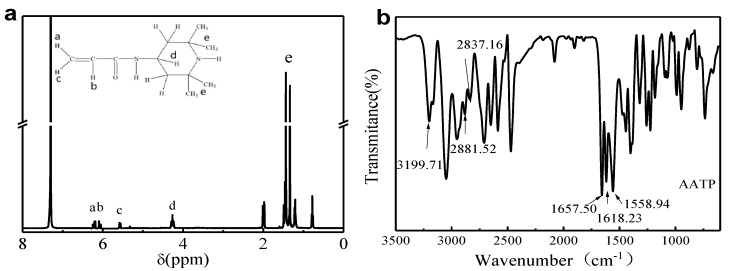
(**a**) ^1^H-NMR spectrum of AATP (**b**) FT-IR spectrum of AATP.

The ^1^H-NMR spectrum of the prepared AATP is displayed in Figure 1a (deuterated chloroform was the solvent). The hydrogen spectroscopy data were as follows: 6.28 (1H, d, vinyl CH_2_), 6.08 (1H, q, vinyl CH), 5.67 (1H, dd, vinyl CH_2_), 5.32 (1H, s, amide NH), 4.37 (1H, d, piperidine CH), 1.97 (2H, dd, piperidine CH_2_), 1.29 (6H, s, 2 × CH_3_), 1.15 (6H, s, 2 × CH_3_), 0.97 (2H, t, piperidine CH_2_). According to the reference [13,26], it was known that the product synthesized was an acrylamide monomer.

The FT-IR spectrum of the prepared AATP is shown in Figure 1b. The characteristic peaks of the AATP were found as follow: 3199.71 cm^−1^ was assigned to the secondary amine N–H stretching vibration peak; 1558.94 cm^−1^ corresponds to the amide NH bending vibration peak; 2953.75 cm^−1^, 2881.56 cm^−1^, and 2873.16 cm^−1^ are, respectively, ascribable to the stretching vibrational peaks of –CH_3_, –CH_2_, and –CH on the piperidine ring; 1653.50 cm^−1^ was attributed to the amide C=O stretching vibration peak; and 1618.23 cm^−1^ is related to the stretching vibration peak of the double bond of C=C.

**Figure 2:**
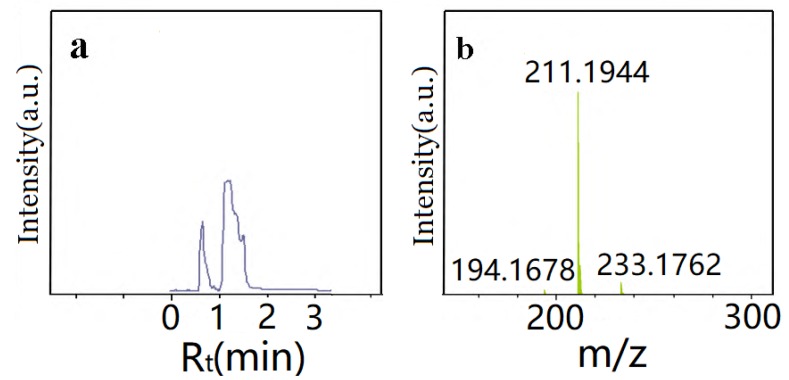
HPLC-MS spectrum of AATP: (**a**) liquid chromatogram and (**b**) mass spectrum. R_t_ represents retention time, m/z represents ratio of mass to charge.

The HPLC-MS spectra of the monomer AATP are shown in Figure 2. The peak at 1.4 min in liquid chromatogram corresponds to AATP (in Figure 2a). The ratio of mass to charge m/z of 211.1944 was assigned to a molecular ion peak [M + 1] (Figure 2b). Since the AATP ion is a cation whose mass was increased by 1 in the mass spectrum, the molecule mass [M] of the AATP should be 210.1944. The theoretical relative molecular mass of AATP is 210.1932. Based the above results of FT-IR, ^1^H-NMR, and HPLC-MS, it was confirmed that the product prepared was the AATP monomer [17].

### 3.2. Structural Characterization of Poly(TAm-co-SSS) and PTAm

#### 3.2.1. ^1^H-NMR Analysis of Poly(AATP-co-SSS) and PAATP

**Figure 3:**
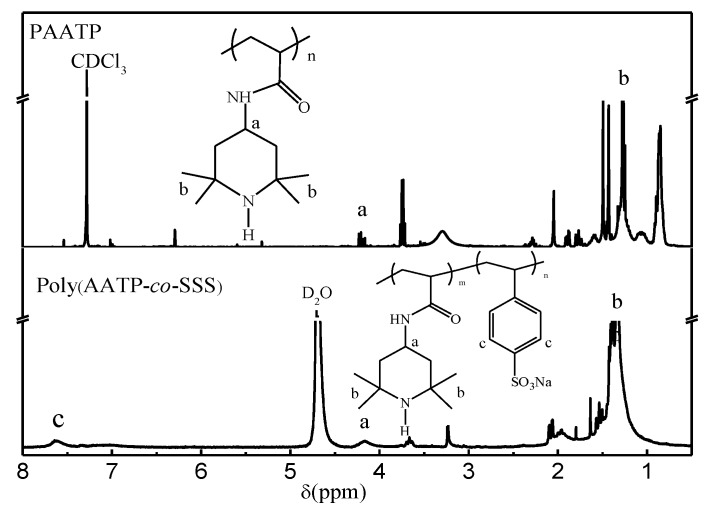
^1^H-NMR spectra of poly(AATP-*co*-SSS) in D_2_O and PAATP in CDCl_2_.

The ^1^H-NMR analyses were carried out to identity the copolymers poly(AATP-*co*-SSS) and PAATP. The characteristic spectra are displayed in Figure 3 as follows. For both poly(AATP-*co*-SSS) and PAATP, the peak at 4.20 ppm was assigned to C–H (a) on piperidine attached to amide group. The broad peak at 1.08 ppm is ascribable to the chemical shifts of four methyl-side groups (b) of piperidine ring. However, the peak of 7.60 ppm which corresponds to hydrogen from the benzene ring (c) was only in the spectrum of poly(AATP-*co*-SSS). Besides, the C=C bonds of AATP and SSS were participating in the polymerization; they became single bonds. That indicates that AATP and SSS were successfully copolymerized to form the polymer poly(AATP-*co*-SSS).

#### 3.2.2. FT-IR Analysis of PTAm, Poly(AATP-co-SSS), and Poly(TAm-co-SSS)

**Figure 4:**
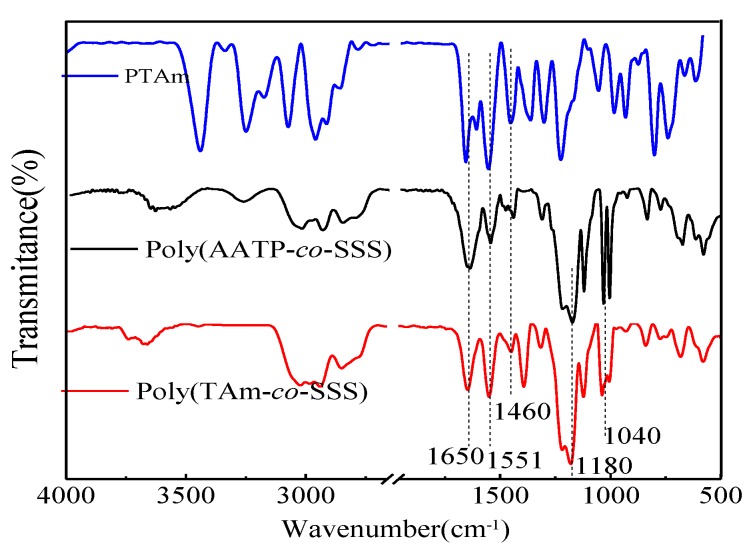
FT-IR spectrum of poly(TAm-*co*-SSS), poly(AATP-*co*-SSS), and PTAm.

The FT-IR characteristic peaks of PTAm, poly(AATP-*co*-SSS), and poly(TAm-*co*-SSS) were found as shown in Figure 4. The peak at 1551 cm^−1^ was assigned to an amide N–H bending vibration peak; 1650 cm^−1^ was attributed to an amide C=O stretching vibration peak; 1460 cm^−1^ was attributed to an N–O stretching vibration peak; 1180 cm^−1^ and 1040 cm^−1^ respectively, correspond to the antisymmetric and symmetric stretching vibration absorption peaks of sulfonate. Compared with the monomer AATP infrared spectrum (Figure 1b), the characteristic peaks of double bonds participating in the polymerization become single bonds of PTAm. From the above analysis, it was found that AATP was polymerized with sodium styrene sulfonate to form a polymerization product poly(AATP-*co*-SSS) [13]. Compared with the poly(AATP-*co*-SSS) spectrum, the absorption peak of the N–O stretching vibration peak became significantly stronger. It resulted from the secondary amine N–H bond in pyridine ring being oxidized to N–O radical. It also proved that the oxidation product poly(TAm-*co*-SSS) was obtained. 

### 3.3. XPS Analysis of Poly(TAm-co-SSS)

**Figure 5:**
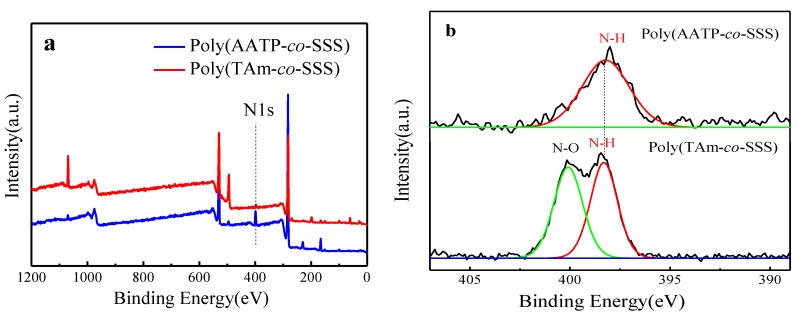
(**a**) XPS broad spectra. (**b**) N1s high resolution spectra of poly(AATP-*co*-SSS) and poly(TAm-*co*-SSS).

As we can see from Figure 5a, the entire nitrogen signal was used to analyze the oxidation of radicals in poly(TAm-*co*-SSS) because the chemical bond N-H in pyridine of the polymer has participated in the oxidation reaction. By X-ray photoelectron spectroscopy analysis of poly(AATP-*co*-SSS) and poly(TAm-*co*-SSS), the 399.8 eV peak in the whole spectrum is assigned to the binding energy of N–H bond in N1s in the polymer [27]. Compared with poly(AATP-*co*-SSS) at this position, the significantly weak absorption intensity of poly(TAm-*co*-SSS) indicates that the N–H of pyridine ring in poly(AATP-*co*-SSS) is oxidized to N–O• in poly(TAm-*co*-SSS) [27]. Furthermore, the high resolution spectrum of N1s in Figure 5b also presents the same result. The N1s resolution spectrum in poly(TAm-*co*-SSS) illustrates two different binding energy peaks including 399.8 eV and 401.0 eV correspond to a protected nitrogen (N–H or CO–NH) and a N–O• functional group, respectively. It is confirmed that the N-H of pyridine ring in poly(AATP-*co*-SSS) is oxidized to N–O• in poly(TAm-*co*-SSS). Moreover, according to the chemical structure of poly(AATP-co-SSS), N–H quantity of pyridine is equal to that of CO–NH. By quantification analysis of N–O and N–H bond of poly(TAm-*co*-SSS), N–H bond of pyridine has almost been oxidized completely because the area of N–O peak is basically equal to N-H peak from CO–NH.

### 3.4. EPR Analysis of Poly(TAm-co-SSS)

**Figure 6:**
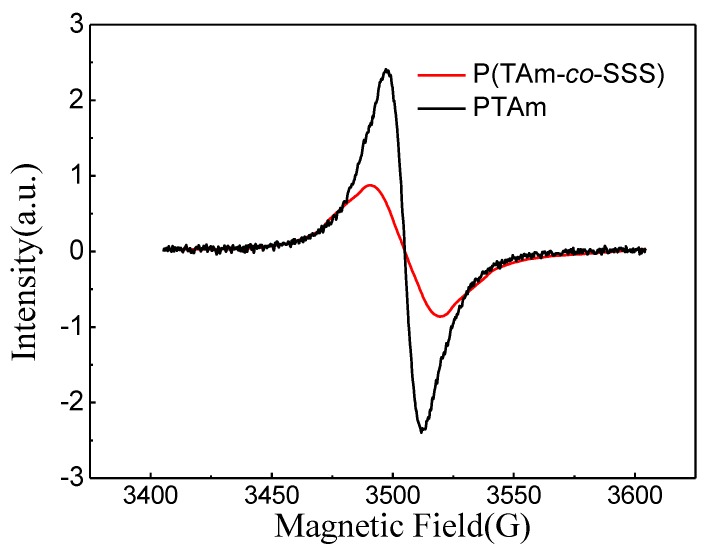
EPR spectra of poly(TAm-*co*-SSS) and PTAm at a concentration of 2 mg/mL in tetrahydrofuran (THF) at room temperature.

The nitroxides polymers are paramagnetic because of stable nitroxides. Thus, EPR was used to determine the N–O• radicals of poly(TAm-*co*-SSS) and PTAm. Figure 6 displays the EPR spectra of poly(TAm-*co*-SSS) and PTAm, respectively. The result shows the hyperfine coupling constant g values of poly(TAm-*co*-SSS) and PTAm are both 2.0047, which was calculated according to the formula hν = gβH corresponding to that of TEMPO (2.0056) [27]. This proves that the nitroxide structure is indeed present in poly(TAm-*co*-SSS). However, its spin-spin exchange and dipole–dipole interactions between molecules or molecules are relatively weakened, which may be due to the TEMPO moiety in the copolymer was partly replaced by sulfonate structure [28]. By quantifying the TEMPO radicals’ intensity, we found that the proportion of SSS section was nearly 25%(mol%). The hyperfine information in the EPR spectrum of the TEMPO radicals in the poly(TAm-*co*-SSS) can be clearly distinguished by the external SSS section. Furthermore, the EPR spectrum of the polymer poly(TAm-*co*-SSS) shows a little broader peak at H = 3505 than that of PTAm. The difference in line width of nitroxyl radicals between poly(TAm-*co*-SSS) and PTAm is mainly due to the constant transition of electrons between two energy levels, which is a dynamic equilibrium process [29].

### 3.5. Thermal Characterization

**Figure 7:**
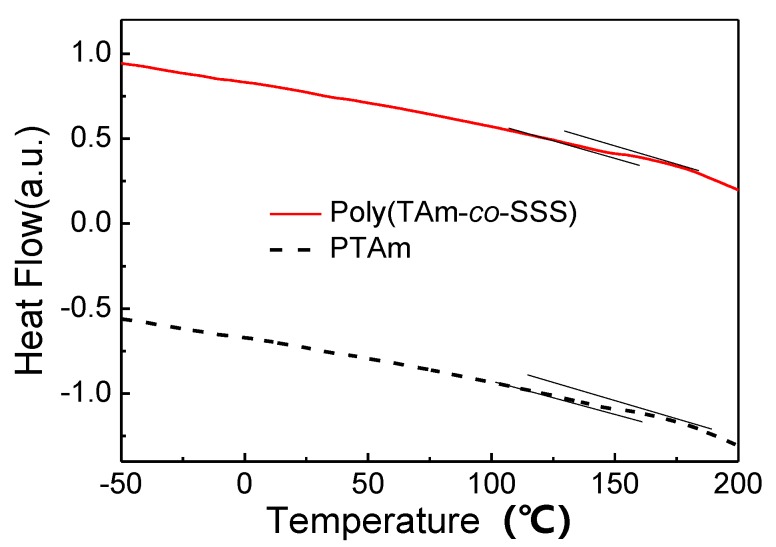
DSC curves of poly(TAm-co-SSS) and PTAm(10 °C/min, nitrogen, second heating).

DSC thermograms corresponding to poly(TAm-*co*-SSS) and PTAm are shown in Figure 7. It can be seen from Figure 7 that the second heating curve shape is similar for both polymers. Only small changes in heat flow were present at around 150 °C. Both polymers having no crystallization or melting endotherm between ambient temperature and 200 °C indicates that the polymers are amorphous, which is highly desirable for lithium ion transport. Thermal behavior observed here is similar with the results described for poly(2,2,6,6-tetramethylpiperidinyloxy methacrylate) (PTMA) in the literature [29]. This result suggests that modification of PTAm with sulfonate does not change thermal behavior.

### 3.6. Electrochemical Performance of Poly(TAm-co-SSS)

#### 3.6.1. Electrochemical Impedance Spectra

**Figure 8:**
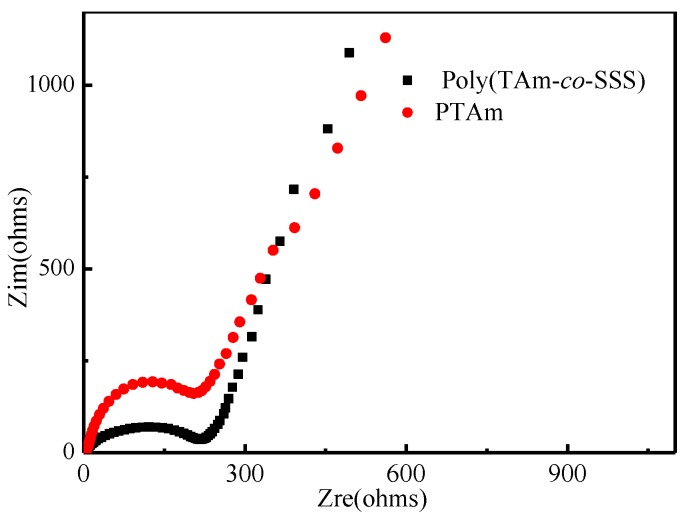
Impedance map of poly(TAm-*co*-SSS) with a scan voltage range of 2.0–4.3 V and a scan rate of 0.5 mV·s^−1^.

It is generally believed that the AC impedance spectrum is composed of a straight line located in the low frequency region and a semicircular curve located in the high frequency region. The body resistance Rb corresponds to the diameter of the semi-circular curve of the high frequency region of the system. Meanwhile, the smaller the diameter is, the smaller the body resistance of the corresponding system is. That is, the conductivity is the higher. Figure 8 shows that the impedance map of poly(TAm-*co*-SSS) exhibits a distinct semicircle in the high frequency range. The radius of the semicircle indicates the impedance of the poly(TAm-*co*-SSS) electrode when it is electrochemically polarized, and presents a straight line at the low frequency band [30]. The semicircle of the AC impedance spectrum gradually shrinks and decreases toward the high frequency, and finally degenerates into a slanted straight line. From the semi-circular curve of the high frequency region in its AC impedance spectrum, the impedance of poly(TAm-*co*-SSS) is 223 Ω, which is almost similar to that of PTAm is 218 Ω. The possible reason is that the nitroxyl radical polymer containing ion holes is activated in the test processing [31]. The doping modification from the sulfonate at the benzene ring affects the internal resistance of the entire electrode [32]. This polymer has good electrical conductivity and can be used as an electrode material for batteries.

#### 3.6.2. Charge–Discharge Performance

**Figure 9:**
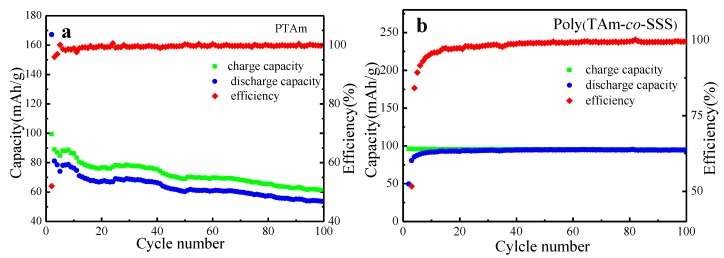
Charge and discharge cycle curves and coulombic efficiency curves of (**a**) PTAm and (**b**) poly(TAm-*co*-SSS) at the current of 100 mA·g^−1^ between 2.0 and 4.3 V in LiPF_6_ EC/DMC (v/v, 1:1) electrolyte versus Li/Li^+^ at room temperature.

Figure 9 presents charge-discharge specific cycle curves and coulombic efficiency curves of (a) PTAm and (b) poly(TAm-*co*-SSS) at the current density of 100 mA·g^−1^. The test conditions of the poly(TAm-*co*-SSS) as a lithium positive electrode material were the same as those of the PTAm. Meantime, the poly(TAm-*co*-SSS) had charge and discharge curves similar to that of the PTAm as a comparative sample. At the initial stabilization cycle, the capacity was slightly attenuated. As the cycle progressed, the capacity of poly(TAm-*co*-SSS) increased to some extent. After 10 charging and discharging cycles, the capacity began to stabilize. That may be because the solid electrolyte interface (SEI) film which formed in the lithium battery hindered the cycle progress of charge and discharge at the first cycle. After that, the carbon structure on the backbone of the nitroxyl radical polymer was gradually activated to be a polymer structure with more electron transfer active sites. It is worth mentioning that the reversible specific capacity of poly(TAm-*co*-SSS) as an active electrode material is 95.7 mAh·g^−1^. After 100 cycles, the specific capacity of poly(TAm-*co*-SSS) was maintained at 95 mAh·g^−1^ (the retention is over 95%), which is higher than of the PTAm. That indicates that the poly(TAm-*co*-SSS) has a better charge-discharge cycling stability than that of the PTAm. Moreover, the first coulombic efficiency (78%) of poly(TAm-*co*-SSS) was higher in comparison with that of PTAm (52%). So the poly(TAm-*co*-SSS) has better electrochemical performance superior to the PTAm. It is may due to the synergistic effect of sulfonate ions group and nitroxyl radical structure which has a great influence on the nitroxyl radical polymer. The sulfonate ions group which was introduced into PTAm not only increases the electron mobility, but also facilitates the transport of lithium ions [33]. PTAm copolymerized with styrene sulfonate utilizes an electro-inactive styrene sulfonate anionic moiety as the self-charge balancing and Li^+^ hosting group to improve exchange of Li^+^ ions [34]. Moreover, PTAm copolymerized with styrene sulfonate has not only hydrophilic but also some hydrophobic nature. Thus, this swollen state of anionic group can facilitate the cation migration in the diffusion front at polymer/electrolyte interface [24]. Consequently, the nitroxyl radical poly(TAm-*co*-SSS) exhibits good electrochemistry Li-ion storage performance.

#### 3.6.3. Change of Morphology

**Figure 10:**
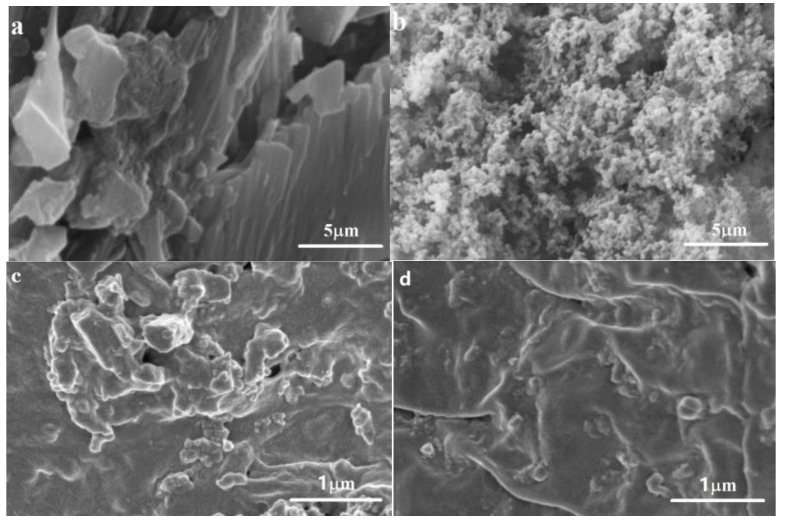
SEM images of (**a**) PTAm; (**b**) poly(TAm-*co*-SSS); and poly(TAm-*co*-SSS) cathode material before (**c**) and after (**d**) cycling tests.

The morphologies of the as-obtained polymers and cathode material before and after the cycling test have also been investigated by SEM. The morphology changes from PTAm to poly(TAm-*co*-SSS) also verifies the stability performance of charge-discharge cycling. As can be seen in Figure 10, the aggregation of (a) PTAm particles is obviously a dense packing plate-like structure. Comparatively, as the PSS-based moieties are introduced into PTAm, the morphology of poly(TAm-*co*-SSS) (b) obviously changes from that of PTAm. A medium-magnification (12,000×) SEM image of poly(TAm-*co*-SSS) clearly shows that the sample has a looser stacking structure. The electrode material with a loose assemble particle and large special surface area is in favor of the utilization of electrode-active material as well as the improvement of the electrochemical properties [22]. SEM images before (c) and after (d) cycling tests at high-magnification (30,000×) are shown in Figure 10. When comparing the surface morphologies of poly(TAm-co-SSS) cathode before and after charge−discharge cycling tests, we also found that the cathode structure nearly maintained its initial state, except for the covered lithium salt and solid electrolyte. Sulfonate sodium as a balance group was introduced to PTAm to achieve both swelling and yet insoluble properties in the aqueous electrolyte solution. During the charging and discharging process, the sulfonate structure can be well maintained without destroying the structure of the nitroxyl radicals, ensuring the cycle stability and safety performances of the materials. All of those are very important to prepare a good cathode material for Li-ion batteries. 

## 4. Conclusions

In this work, a paradigm was extended to the realm of radical polymers through the intentional addition of ion-bearing repeat units to the macromolecular architecture of the radical polymer PTAm. As its key monomer, 4-acrylamido-2,2,6,6-tetramethylpiperidine was prepared using 4-amino-2,2,6,6-tetramethyl-piperidine and acryloyl chloride. The AATP was characterized by ^1^H-NMR, FT-IR, and HPLC-MS. The white powder poly(AATP-*co*-SSS) was obtained by free radical polymerization with sodium styrene sulfonate (SSS) and AATP. After oxidation, a faint yellow solid poly(TAm-*co*-SSS) was formed and characterized by FT-IR and XPS. The structure confirms that the product synthesized was the target product. By performing a button cell assembly on the synthesized radical polymer poly(TAm-*co*-SSS), the capacity and electrochemical performance tests were carried out. Adjusting pendant groups and medium crossing structure can enhance the transport ability and improve the charge-discharge cycling stability. The TEMPO-substituted polyacrylamide bearing sulfonate sodium pendant is expected to be used as an organic electrode material. It provides a vital pathway by which to optimize next-generation radical polymer designs.
